# Crystal structure, DFT study and Hirshfeld surface analysis of 1-nonyl-2,3-di­hydro-1*H*-indole-2,3-dione

**DOI:** 10.1107/S2056989019009691

**Published:** 2019-07-12

**Authors:** Ibtissam Rayni, Youness El Bakri, Chin-Hung Lai, Jihad Sebhaoui, El Mokhtar Essassi, Joel T. Mague

**Affiliations:** a Laboratoire de Chimie Organique Hétérocyclique, Centre de Recherche des Sciences des Médicaments, URAC 21, Pôle de Compétence Pharmacochimie, Av Ibn Battouta, BP 1014, Faculté des Sciences, Université Mohammed V, Rabat, Morocco; b Organic Chemistry Department, Faculty of Science, RUDN University, Miklukho-Maklaya St. 6, 117198 Moscow, Russian Federation; c Department of Medical Applied Chemistry, Chung Shan Medical University, Taichung 40241, Taiwan; d Department of Medical Education, Chung Shan Medical University Hospital, 402 Taichung, Taiwan; e Department of Chemistry, Tulane University, New Orleans, LA 70118, USA

**Keywords:** crystal structure, di­hydro­indole­dione, hydrogen bond, micelle, π-stacking

## Abstract

In the title mol­ecule, C_17_H_23_NO_2_, the di­hydro­indole portion is planar (r.m.s. deviation = 0.0157 Å) and the nonyl substituent is in an ‘extended’ conformation. In the crystal, the nonyl chains inter­calate and the di­hydro­indole­dione units are associated through C—H⋯O hydrogen bonds to form micellar blocks. Based on the Hirshfeld surface analysis, the most important inter­molecular inter­action is the H⋯H inter­action.

## Abstract 2

The di­hydro­indole portion is planar and the nonyl substituent is in an ‘extended’ conformation. In the crystal, the nonyl chains inter­calate aided by pairwise C—H⋯O hydrogen bonds and the di­hydro­indole­dione units are associated through additional C—H⋯O hydrogen bonds to form micellar blocks. The blocks are linked through π-stacking inter­actions between the six-membered rings of the di­hydro­indole units.

## Chemical context   

Indoline-2,3-dione or indole-*1H*-2,3-dione, commonly known as isatin, is a well-known natural product found in plants of genus *Isatis* and in *Couropita guianancis aubl* (Da Silva *et al.*, 2001 ▸). It has also been isolated as a metabolic derivative of adrenaline in humans (Almeida *et al.*, 2010 ▸). It was first obtained as an oxidation product of indigo in the early 19th century, and its current structure was proposed by Kekulé (1869 ▸). Isatin is a core constituent of many alkaloids (Trost *et al.*, 2009 ▸) and drugs (Aboul-Fadl *et al.*, 2010 ▸) as well as dyes (Doménech *et al.*, 2009 ▸), pesticides and analytical reagents. Isatin derivatives possess diverse activities such as anti­bacterial (Kassab *et al.*, 2010 ▸), anti­viral (Jarrahpour *et al.*, 2007 ▸), anti-HIV (Sriram *et al.*, 2006 ▸), anti­cancer (Gürsoy *et al.*, 2003 ▸) and anti-inflammatory (Sridhar *et al.*, 2001 ▸) activities. As a continuation of our research work devoted to the development of isatin derivatives (Ben-Yahia *et al.*, 2018 ▸; Rayni *et al.*, 2019 ▸), we report in this work the synthesis and the Hirshfeld surface analysis of a new indoline-2,3-dione derivative obtained by the action of nonyl bromide on isatin under phase-transfer catalysis conditions.
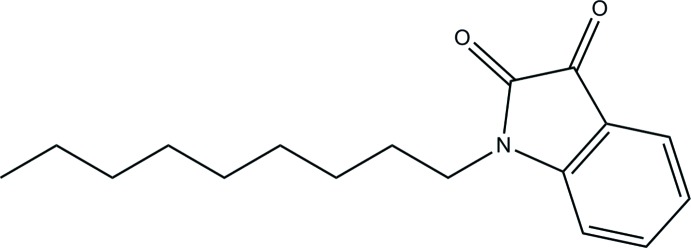


## Structural commentary   

**Figure 1:**
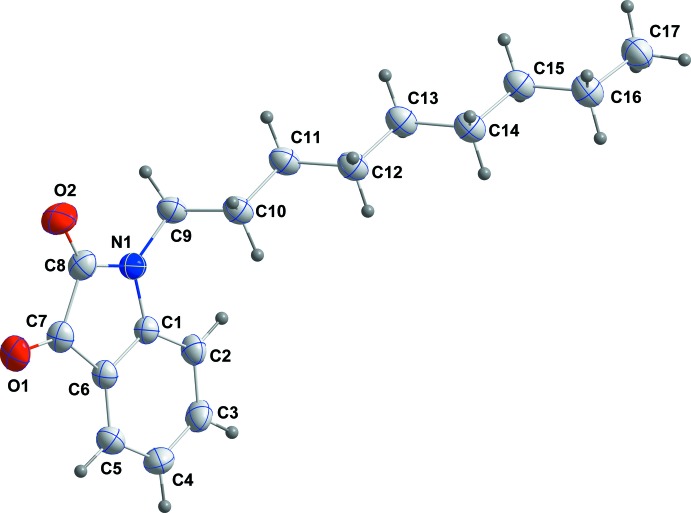
The title mol­ecule with the labelling scheme and 50% probability ellipsoids.

The mol­ecular structure of the title compound is shown in Fig. 1 ▸. The di­hydro­indole skeleton is planar to within 0.0286 (8) Å (r.m.s. deviation of the fitted atoms = 0.0157 Å) with Cl being the furthest from the mean plane. The nonyl chain is in an ‘extended’ conformation and is well out of the mean plane of the di­hydro­indole unit, as indicated by the C1—N1—C9—C10 torsion angle of −69.94 (12)°.

## Supra­molecular features   

**Figure 2:**
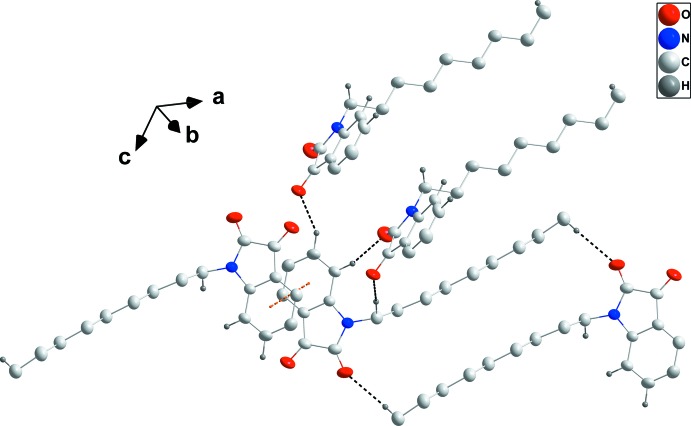
Detail of the inter­molecular inter­actions. C—H⋯O hydrogen bonds and π-stacking inter­actions are shown, respectively, by black and orange dashed lines. H atoms not involved in hydrogen bonds are omitted for clarity.

In the crystal, the mol­ecules pack in a typical micellar manner with the di­hydro­indoldione head groups associated through C2—H2⋯O2^i^, C3—H3⋯O1^ii^ and C9—H9*B*⋯O1^i^ hydrogen bonds (Table 1 ▸) and the nonyl ‘tails’ inter­calating and aided by paired C17—H17*B*⋯O2^iii^ hydrogen bonds (Table 1 ▸ and Fig. 2 ▸). The micellar blocks are associated through π-stacking inter­actions between inversion-related C1–C6 rings [centroid–centroid distance = 3.6470 (7) Å; Figs. 2 ▸ and 3 ▸].

## Database survey   

A search of the Cambridge Crystallographic Database (Version 5.40 updated to April 2019; Groom *et al.*, 2016 ▸) provided structures of 11 derivatives of the di­hydro­indole-2,3-dione skeleton having a saturated carbon chain of at least three atoms bound to nitro­gen. Thus, in place of the *n*-nonyl chain (*R*) in the title compound, there are ones with *R* = 3-bromo­propyl (AKOBIN; Qachchachi *et al.*, 2016*a* ▸), *n*-propyl (AKOCOU; Qachchachi *et al.*, 2016*b* ▸), *n*-octyl (CIQDOX; Qachchachi *et al.*, 2013 ▸), 2,3-di­benzoyl­ethane (FUBLIZ; Žari *et al.*, 2015 ▸), *n*-dodecyl (GITTEK; Qachchachi *et al.*, 2014*a* ▸), cyclo­pentyl (JOWSOF; Mironova *et al.*, 2015 ▸), 3-carb­oxy­methyl­propane (JOWSUL; Mironova *et al.*, 2015 ▸), 2-cyano­ethane (LIVSIU; Qachchachi *et al.*, 2014*b* ▸), *n*-tetra­decyl (TUPSIH; Mamari *et al.*, 2010*a* ▸) and *n*-decyl (TUPSON; Mamari *et al.*, 2010*b* ▸). In addition, there is one structure with two di­hydro­indole-2,3-dione moieties connected by a –(CH_2_)_6_– linkage (OJIGOF; Qachchachi *et al.*, 2016*c* ▸). In all of these compounds, the di­hydro­indole-2,3-dione skeleton is planar and the first two carbon atoms from the nitro­gen are rotated so that the N–C–C plane is nearly perpendicular to the plane of the di­hydro­indole-2,3-dione. Additionally, the C—C distances corresponding to the C7—C8 distance in the title structure [1.5554 (15) Å] are in the range 1.543 (4)–1.563 (6) Å. Generally, the carbon chains are in an ‘extended’ conformation.

## Calculation of the electronic structure   

**Figure 4:**
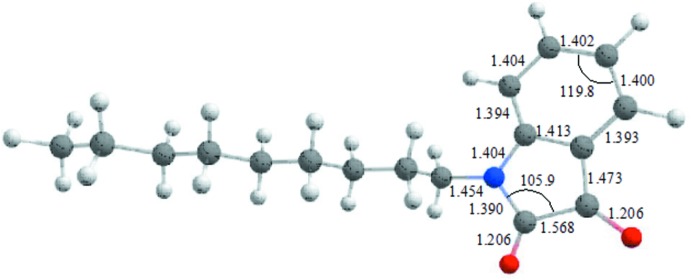
The B3LYP-optimized geometry of the title compound (bond lengths in Å, bond angles in °; carbon in gray, nitro­gen in blue, oxygen in red and hydrogen in white). please improve resolution

The structure in the gas phase of the title compound was optimized by means of density functional theory. The DFT calculation was performed using the hybrid B3LYP method, which is based on the idea of Becke and considers a mixture of the exact (HF) and DFT exchange utilizing the B3 functional, together with the LYP correlation functional (Becke, 1993 ▸; Lee *et al.*, 1988 ▸; Miehlich *et al.*, 1989 ▸). The B3LYP calculation was performed in conjunction with the def2-SVP basis set (Weigend & Ahlrichs, 2005 ▸). After obtaining the converged geometry, the harmonic vibrational frequencies were calculated on the same theoretical level to confirm that the number of imaginary frequencies is zero for the stationary point. Both the geometry optimization and the harmonic vibrational frequency analysis of the title compound were performed using the *Gaussian 16* program (Frisch *et al.*, 2016 ▸). The result of the B3LYP geometry optimization for the title compound (shown in Fig. 4 ▸) was compared to that of the crystallographic study with selected geometric parameters for the gas-phase and solid-phase structures summarized in Table 2 ▸. This shows that there is a clear discrepancy between the B3LYP-optimized geometry and the X-ray geometry. To qu­antify this, the *openBabel* program was then used to convert the experimental CIF file to a *Gaussian* .gjf input file (O’Boyle *et al.*, 2011 ▸). The structure compared built in the *ChemCraft* program (graphical software for visualization of quantum chemistry computations; https://www.chemcraftprog.com) was finally used to obtain a weighted r.m.s. deviation of 0.5808 Å with r.m.s.d. values of of 0.6297, 0.5213, 0.2231, and 0.5977 Å, respectively, for the H, C, N and O atoms.

## Hirshfeld surface analysis   

**Figure 5:**
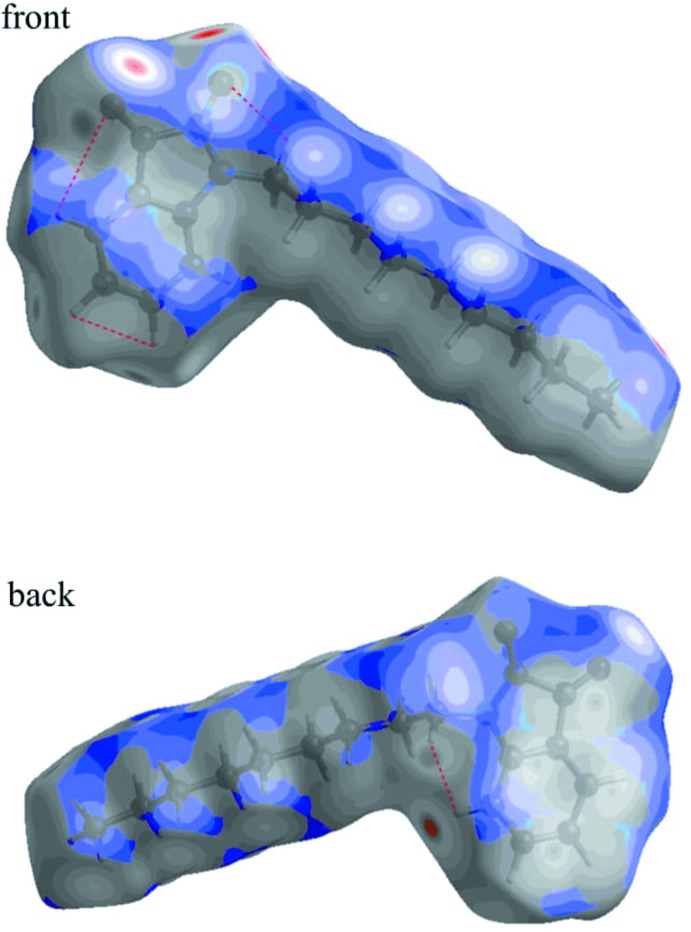
The *d*_norm_ Hirshfeld surface of the title compound (red: negative, white: zero, blue: positive; scale: −0.2101 to 1.3375 a.u.).

**Figure 6:**
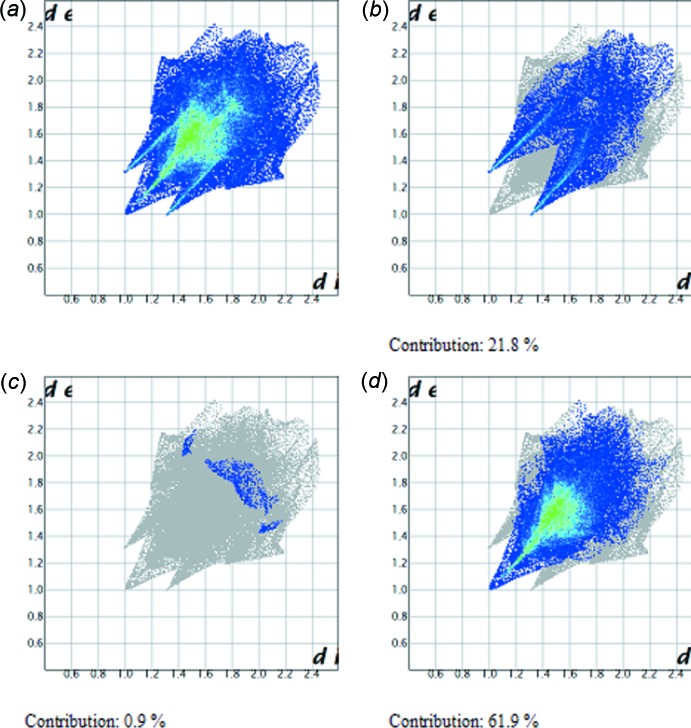
Fingerprint plots for the title compound: (*a*) full and delineated into (*b*) H⋯O/O⋯H, (*c*) H⋯N/N⋯H and (*d*) H⋯H contacts.

Both the definition of a mol­ecule in a condensed phase and the recognition of distinct entities in mol­ecular liquids and crystals are fundamental concepts in chemistry. Based on Hirshfeld’s partitioning scheme, Spackman *et al.* (1997 ▸) proposed a method to divide the electron distribution in a crystalline phase into mol­ecular fragments (Spackman & Byrom, 1997 ▸; McKinnon *et al.*, 2004 ▸; Spackman & Jayatilaka, 2009 ▸). Their proposed method partitioned the crystal into regions where the electron distribution of a sum of spherical atoms for the mol­ecule dominates over the corresponding sum of the crystal. In this study, the Hirshfeld surface analysis of the title compound was performed utilizing the *CrystalExplorer* program (Turner *et al.*, 2017 ▸). The standard resolution mol­ecular Hirshfeld surface (*d*_norm_) of the title compound is depicted in Fig. 5 ▸. This surface can be used to identify very close inter­molecular inter­actions. The value of *d*_norm_ is negative (positive) when inter­molecular contacts are shorter (longer) than the van der Waals radii. The *d*_norm_ value is mapped onto the Hirshfeld surface using red, white or blue colours. The red regions represent closer contacts with a negative *d*_norm_ value while the blue regions represent longer contacts with a positive *d*_norm_ value. The white regions represent contacts equal to the van der Waals separation and have a *d*_norm_ value of zero. As depicted in Fig. 5 ▸, important inter­actions in the title compound are H⋯O and H⋯N hydrogen bonds. The two-dimensional fingerprint plots (Fig. 6 ▸) highlight particular atom-pair contacts and enable the separation of contributions from different inter­action types that overlap in the full fingerprint. The most important inter­actions involving the hydrogen atoms in the title compound are the H⋯H contactso. The H⋯H, H⋯O/O⋯H and H⋯N/N⋯H contacts make contribututions of 61.9, 21.8 and 0.9%, respectively, to the Hirshfeld surface.

## Synthesis and crystallization   

To a solution of isatin (0.5 g, 3.4 mmol) dissolved in 25 ml of *N*,*N*-di­methyl­formamide, 1-bromo­octane (0.7 ml, 3.4 mmol), potassium carbonate (0.61 g, 4.4 mmol) and a catalytic amount of tetra-*n*-butyl­ammonium bromide (0.1 g, 0.4 mmol) were added. The mixture was stirred for 48 h and the reaction monitored by thin layer chromatography. The mixture was filtered and the solvent removed under vacuum. The solid obtained was recrystallized from ethanol to afford the title compound as orange–red crystals.

## Refinement   

Crystal data, data collection and structure refinement details are summarized in Table 3 ▸.

## Supplementary Material

Crystal structure: contains datablock(s) global, I. DOI: 10.1107/S2056989019009691/vm2219sup1.cif

Structure factors: contains datablock(s) I. DOI: 10.1107/S2056989019009691/vm2219Isup2.hkl

Supporting information file. DOI: 10.1107/S2056989019009691/vm2219Isup3.cdx

Supporting information file. DOI: 10.1107/S2056989019009691/vm2219Isup4.cml

CCDC reference: 1938997

Additional supporting information:  crystallographic information; 3D view; checkCIF report

## Figures and Tables

**Figure 3:**
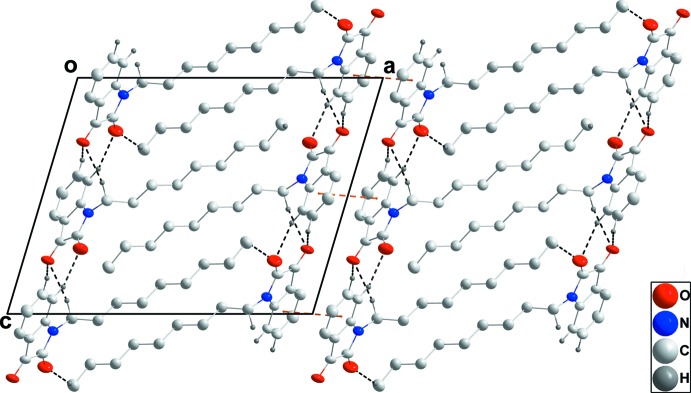
Packing viewed along the *b*-axis direction with inter­molecular inter­actions depicted as in Fig. 2 ▸. H atoms not involved in hydrogen bonds are omitted for clarity.

**Table 1:** Hydrogen-bond geometry (Å, °)

*D*—H⋯*A*	*D*—H	H⋯*A*	*D*⋯*A*	*D*—H⋯*A*
C2—H2⋯O2^i^	0.992 (13)	2.412 (13)	3.3737 (13)	163.3 (10)
C3—H3⋯O1^ii^	0.997 (14)	2.454 (15)	3.2734 (14)	139.0 (11)
C9—H9*B*⋯O1^i^	0.994 (13)	2.546 (13)	3.5012 (13)	161.0 (10)
C17—H17*B*⋯O2^iii^	0.98 (2)	2.49 (2)	3.3941 (17)	153.3 (15)

Symmetry codes: (i) 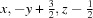; (ii) 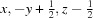; (iii) 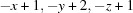.

**Table 2:** The B3LYP-optimized and X-ray structural parameters (Å, °) for the title compound

	B3LYP	X-ray
C1—C2	1.394	1.3806 (13)
C2—C3	1.404	1.3899 (16)
C3—C4	1.402	1.3868 (16)
C4—C5	1.400	1.3871 (16)
C5—C6	1.393	1.3862 (15)
C6—C7	1.473	1.4599 (13)
C6—C1	1.413	1.4009 (13)
C7—C8	1.568	1.5554 (15)
C8—N1	1.390	1.3603 (13)
N1—C1	1.404	1.4127 (13)
C7—O1	1.206	1.2126 (12)
C8—O2	1.206	1.2106 (13)
N1—C9	1.454	1.4606 (13)
N1—C8—C7	105.9	106.20 (8)

**Table 3:** Experimental details

Crystal data	
Chemical formula	C_17_H_23_NO_2_
*M*_r_	273.36
Crystal system, space group	Monoclinic, *P*2_1_/*c*
Temperature (K)	150
*a*, *b*, *c* (Å)	16.2512 (4), 7.6859 (2), 13.0989 (3)
β (°)	106.640 (1)
*V* (Å^3^)	1567.60 (7)
*Z*	4
Radiation type	Cu *K*α
μ (mm^−1^)	0.59
Crystal size (mm)	0.24 × 0.20 × 0.14

Data collection	
Diffractometer	Bruker D8 VENTURE PHOTON 100 CMOS
Absorption correction	Multi-scan (*SADABS*; Krause *et al.*, 2015 ▸)
*T*_min_, *T*_max_	0.82, 0.92
No. of measured, independent and observed [*I* > 2σ(*I*)] reflections	11594, 3128, 2879
*R*_int_	0.029
(sin θ/λ)_max_ (Å^−1^)	0.625

Refinement	
*R*[*F* ^2^ > 2σ(*F* ^2^)], *wR*(*F* ^2^), *S*	0.035, 0.096, 1.05
No. of reflections	3128
No. of parameters	274
H-atom treatment	All H-atom parameters refined
Δρ_max_, Δρ_min_ (e Å^−3^)	0.22, −0.14

Computer programs: *APEX3* and *SAINT* (Bruker, 2016 ▸), *SHELXT* (Sheldrick, 2015*a* ▸), *SHELXL2018* (Sheldrick, 2015*b* ▸), *DIAMOND* (Brandenburg & Putz, 2012 ▸) and *SHELXTL* (Sheldrick, 2008).

## supplementary crystallographic information

### Crystal data

**Table:**

C_17_H_23_NO_2_	*F*(000) = 592
*M**_r_* = 273.36	*D*_x_ = 1.158 Mg m^−^^3^
Monoclinic, *P*2_1_/*c*	Cu *K*α radiation, λ = 1.54178 Å
*a* = 16.2512 (4) Å	Cell parameters from 9962 reflections
*b* = 7.6859 (2) Å	θ = 5.1–74.4°
*c* = 13.0989 (3) Å	µ = 0.59 mm^−^^1^
β = 106.640 (1)°	*T* = 150 K
*V* = 1567.60 (7) Å^3^	Block, orange-red
*Z* = 4	0.24 × 0.20 × 0.14 mm

### Data collection

**Table:**

Bruker D8 VENTURE PHOTON 100 CMOS diffractometer	3128 independent reflections
Radiation source: INCOATEC IµS micro–focus source	2879 reflections with *I* > 2σ(*I*)
Mirror monochromator	*R*_int_ = 0.029
Detector resolution: 10.4167 pixels mm^-1^	θ_max_ = 74.4°, θ_min_ = 6.4°
ω scans	*h* = −18→19
Absorption correction: multi-scan (*SADABS*; Krause *et al.*, 2015)	*k* = −9→8
*T*_min_ = 0.82, *T*_max_ = 0.92	*l* = −16→15
11594 measured reflections	

### Refinement

**Table:**

Refinement on *F*^2^	Secondary atom site location: difference Fourier map
Least-squares matrix: full	Hydrogen site location: difference Fourier map
*R*[*F*^2^ > 2σ(*F*^2^)] = 0.035	All H-atom parameters refined
*wR*(*F*^2^) = 0.096	*w* = 1/[σ^2^(*F*_o_^2^) + (0.0474*P*)^2^ + 0.2775*P*] where *P* = (*F*_o_^2^ + 2*F*_c_^2^)/3
*S* = 1.05	(Δ/σ)_max_ = 0.001
3128 reflections	Δρ_max_ = 0.22 e Å^−^^3^
274 parameters	Δρ_min_ = −0.14 e Å^−^^3^
0 restraints	Extinction correction: *SHELXL2018* (Sheldrick, 2015*b*), Fc^*^=kFc[1+0.001xFc^2^λ^3^/sin(2θ)]^-1/4^
Primary atom site location: structure-invariant direct methods	Extinction coefficient: 0.0114 (9)

### Special details

**Table:**

Geometry. All esds (except the esd in the dihedral angle between two l.s. planes) are estimated using the full covariance matrix. The cell esds are taken into account individually in the estimation of esds in distances, angles and torsion angles; correlations between esds in cell parameters are only used when they are defined by crystal symmetry. An approximate (isotropic) treatment of cell esds is used for estimating esds involving l.s. planes.
Refinement. Refinement of F^2^ against ALL reflections. The weighted R-factor wR and goodness of fit S are based on F^2^, conventional R-factors R are based on F, with F set to zero for negative F^2^. The threshold expression of F^2^ > 2sigma(F^2^) is used only for calculating R-factors(gt) etc. and is not relevant to the choice of reflections for refinement. R-factors based on F^2^ are statistically about twice as large as those based on F, and R- factors based on ALL data will be even larger.

### Fractional atomic coordinates and isotropic or equivalent isotropic displacement parameters (Å^2^)

**Table:**

	*x*	*y*	*z*	*U*_iso_*/*U*_eq_	
O1	0.08032 (5)	0.55941 (11)	0.77294 (5)	0.0450 (2)	
O2	0.17619 (6)	0.85773 (11)	0.72453 (7)	0.0545 (3)	
N1	0.16664 (5)	0.69895 (11)	0.57210 (6)	0.0326 (2)	
C1	0.13290 (6)	0.53470 (13)	0.53302 (7)	0.0295 (2)	
C2	0.13301 (6)	0.45892 (14)	0.43750 (8)	0.0351 (2)	
H2	0.1570 (8)	0.5200 (17)	0.3857 (10)	0.045 (3)*	
C3	0.09707 (7)	0.29390 (15)	0.41701 (9)	0.0418 (3)	
H3	0.0954 (9)	0.2350 (19)	0.3486 (11)	0.052 (4)*	
C4	0.06188 (8)	0.20883 (15)	0.48797 (10)	0.0443 (3)	
H4	0.0372 (9)	0.094 (2)	0.4688 (11)	0.059 (4)*	
C5	0.05985 (7)	0.28781 (14)	0.58244 (9)	0.0389 (3)	
H5	0.0330 (9)	0.2317 (19)	0.6319 (11)	0.052 (4)*	
C6	0.09576 (6)	0.45211 (13)	0.60433 (7)	0.0314 (2)	
C7	0.10401 (6)	0.57137 (14)	0.69345 (7)	0.0341 (2)	
C8	0.15339 (7)	0.73151 (14)	0.66825 (8)	0.0365 (2)	
C9	0.21591 (7)	0.80831 (14)	0.51971 (9)	0.0363 (2)	
H9A	0.2192 (8)	0.9233 (18)	0.5546 (10)	0.046 (3)*	
H9B	0.1825 (8)	0.8201 (16)	0.4435 (10)	0.039 (3)*	
C10	0.30417 (7)	0.73223 (15)	0.52814 (9)	0.0373 (2)	
H10A	0.3406 (9)	0.7374 (18)	0.6044 (12)	0.051 (4)*	
H10B	0.2979 (8)	0.6044 (19)	0.5107 (10)	0.044 (3)*	
C11	0.34813 (7)	0.82187 (15)	0.45444 (9)	0.0386 (3)	
H11A	0.3578 (9)	0.946 (2)	0.4751 (11)	0.051 (4)*	
H11B	0.3094 (8)	0.8199 (17)	0.3812 (11)	0.046 (3)*	
C12	0.43166 (7)	0.73437 (15)	0.45297 (9)	0.0395 (3)	
H12A	0.4735 (9)	0.7357 (19)	0.5258 (11)	0.052 (4)*	
H12B	0.4207 (9)	0.611 (2)	0.4372 (11)	0.052 (4)*	
C13	0.47345 (7)	0.81285 (16)	0.37372 (9)	0.0405 (3)	
H13A	0.4834 (9)	0.940 (2)	0.3898 (11)	0.056 (4)*	
H13B	0.4323 (9)	0.8053 (17)	0.3010 (11)	0.049 (4)*	
C14	0.55597 (7)	0.72275 (16)	0.37127 (9)	0.0405 (3)	
H14A	0.5991 (9)	0.7303 (19)	0.4437 (12)	0.056 (4)*	
H14B	0.5448 (9)	0.595 (2)	0.3575 (12)	0.057 (4)*	
C15	0.59615 (7)	0.79507 (16)	0.28902 (9)	0.0403 (3)	
H15A	0.6056 (9)	0.921 (2)	0.3018 (11)	0.059 (4)*	
H15B	0.5542 (9)	0.7861 (17)	0.2165 (11)	0.048 (3)*	
C16	0.67845 (8)	0.70487 (17)	0.28662 (10)	0.0448 (3)	
H16A	0.7217 (10)	0.725 (2)	0.3567 (13)	0.064 (4)*	
H16B	0.6688 (10)	0.575 (2)	0.2808 (13)	0.068 (4)*	
C17	0.71335 (9)	0.76729 (19)	0.19762 (11)	0.0494 (3)	
H17A	0.6695 (11)	0.753 (2)	0.1291 (14)	0.070 (5)*	
H17B	0.7272 (12)	0.892 (3)	0.2051 (14)	0.081 (5)*	
H17C	0.7687 (12)	0.699 (2)	0.1952 (14)	0.076 (5)*	

### Atomic displacement parameters (Å^2^)

**Table:**

	*U*^11^	*U*^22^	*U*^33^	*U*^12^	*U*^13^	*U*^23^
O1	0.0578 (5)	0.0554 (5)	0.0250 (4)	0.0034 (4)	0.0169 (3)	0.0028 (3)
O2	0.0739 (6)	0.0483 (5)	0.0427 (5)	−0.0124 (4)	0.0191 (4)	−0.0180 (4)
N1	0.0383 (5)	0.0331 (4)	0.0272 (4)	−0.0027 (3)	0.0110 (3)	−0.0016 (3)
C1	0.0299 (5)	0.0327 (5)	0.0254 (4)	0.0014 (4)	0.0072 (3)	−0.0003 (3)
C2	0.0360 (5)	0.0430 (6)	0.0286 (5)	−0.0006 (4)	0.0129 (4)	−0.0041 (4)
C3	0.0421 (6)	0.0468 (6)	0.0384 (6)	−0.0016 (5)	0.0148 (4)	−0.0141 (5)
C4	0.0477 (6)	0.0365 (6)	0.0510 (7)	−0.0068 (5)	0.0178 (5)	−0.0096 (5)
C5	0.0418 (6)	0.0376 (6)	0.0394 (6)	−0.0019 (4)	0.0149 (4)	0.0029 (4)
C6	0.0338 (5)	0.0353 (5)	0.0251 (4)	0.0021 (4)	0.0086 (3)	0.0016 (4)
C7	0.0385 (5)	0.0408 (5)	0.0222 (4)	0.0039 (4)	0.0076 (4)	0.0020 (4)
C8	0.0426 (6)	0.0390 (5)	0.0270 (5)	0.0006 (4)	0.0083 (4)	−0.0035 (4)
C9	0.0394 (6)	0.0341 (5)	0.0360 (5)	−0.0014 (4)	0.0113 (4)	0.0058 (4)
C10	0.0365 (5)	0.0390 (6)	0.0357 (5)	−0.0010 (4)	0.0092 (4)	0.0072 (4)
C11	0.0375 (5)	0.0393 (6)	0.0381 (5)	−0.0022 (4)	0.0095 (4)	0.0080 (4)
C12	0.0367 (6)	0.0422 (6)	0.0387 (6)	−0.0016 (4)	0.0094 (4)	0.0068 (4)
C13	0.0371 (6)	0.0461 (6)	0.0370 (6)	−0.0011 (4)	0.0086 (4)	0.0070 (5)
C14	0.0368 (6)	0.0457 (6)	0.0378 (6)	−0.0014 (4)	0.0088 (4)	0.0058 (4)
C15	0.0384 (6)	0.0450 (6)	0.0364 (5)	−0.0016 (4)	0.0090 (4)	0.0052 (4)
C16	0.0388 (6)	0.0523 (7)	0.0435 (6)	−0.0006 (5)	0.0118 (5)	0.0066 (5)
C17	0.0496 (7)	0.0541 (7)	0.0486 (7)	−0.0067 (6)	0.0205 (6)	−0.0001 (6)

### Geometric parameters (Å, º)

**Table:**

O1—C7	1.2126 (12)	C10—H10B	1.007 (14)
O2—C8	1.2106 (13)	C11—C12	1.5200 (16)
N1—C8	1.3603 (13)	C11—H11A	0.989 (15)
N1—C1	1.4127 (13)	C11—H11B	0.986 (14)
N1—C9	1.4606 (13)	C12—C13	1.5186 (15)
C1—C2	1.3806 (13)	C12—H12A	1.000 (14)
C1—C6	1.4009 (13)	C12—H12B	0.979 (16)
C2—C3	1.3899 (16)	C13—C14	1.5178 (16)
C2—H2	0.992 (13)	C13—H13A	1.007 (16)
C3—C4	1.3868 (16)	C13—H13B	0.997 (14)
C3—H3	0.997 (14)	C14—C15	1.5163 (15)
C4—C5	1.3871 (16)	C14—H14A	1.007 (15)
C4—H4	0.971 (16)	C14—H14B	1.002 (16)
C5—C6	1.3862 (15)	C15—C16	1.5147 (17)
C5—H5	0.980 (14)	C15—H15A	0.986 (17)
C6—C7	1.4599 (13)	C15—H15B	1.000 (14)
C7—C8	1.5554 (15)	C16—C17	1.5132 (16)
C9—C10	1.5233 (15)	C16—H16A	0.995 (17)
C9—H9A	0.989 (14)	C16—H16B	1.011 (18)
C9—H9B	0.994 (13)	C17—H17A	0.979 (18)
C10—C11	1.5204 (14)	C17—H17B	0.98 (2)
C10—H10A	1.006 (15)	C17—H17C	1.050 (18)
			
C8—N1—C1	110.61 (8)	C10—C11—H11A	109.2 (8)
C8—N1—C9	125.63 (9)	C12—C11—H11B	107.8 (8)
C1—N1—C9	123.52 (8)	C10—C11—H11B	109.0 (8)
C2—C1—C6	121.72 (9)	H11A—C11—H11B	106.7 (11)
C2—C1—N1	127.18 (9)	C13—C12—C11	114.13 (9)
C6—C1—N1	111.09 (8)	C13—C12—H12A	109.5 (8)
C1—C2—C3	116.77 (9)	C11—C12—H12A	110.4 (8)
C1—C2—H2	121.5 (8)	C13—C12—H12B	109.4 (8)
C3—C2—H2	121.7 (8)	C11—C12—H12B	108.8 (8)
C4—C3—C2	122.14 (10)	H12A—C12—H12B	104.1 (12)
C4—C3—H3	118.6 (8)	C14—C13—C12	113.83 (9)
C2—C3—H3	119.3 (8)	C14—C13—H13A	111.1 (8)
C3—C4—C5	120.75 (10)	C12—C13—H13A	108.6 (8)
C3—C4—H4	118.3 (8)	C14—C13—H13B	108.0 (8)
C5—C4—H4	120.9 (8)	C12—C13—H13B	108.7 (8)
C6—C5—C4	117.84 (10)	H13A—C13—H13B	106.3 (11)
C6—C5—H5	120.4 (8)	C15—C14—C13	114.24 (9)
C4—C5—H5	121.8 (9)	C15—C14—H14A	108.8 (8)
C5—C6—C1	120.73 (9)	C13—C14—H14A	109.6 (8)
C5—C6—C7	132.39 (9)	C15—C14—H14B	108.9 (8)
C1—C6—C7	106.87 (8)	C13—C14—H14B	109.5 (8)
O1—C7—C6	131.29 (10)	H14A—C14—H14B	105.5 (12)
O1—C7—C8	123.52 (9)	C16—C15—C14	114.17 (10)
C6—C7—C8	105.19 (8)	C16—C15—H15A	110.9 (9)
O2—C8—N1	127.53 (10)	C14—C15—H15A	108.5 (8)
O2—C8—C7	126.26 (9)	C16—C15—H15B	108.3 (8)
N1—C8—C7	106.20 (8)	C14—C15—H15B	109.5 (8)
N1—C9—C10	112.12 (8)	H15A—C15—H15B	105.0 (11)
N1—C9—H9A	105.1 (8)	C17—C16—C15	113.51 (10)
C10—C9—H9A	112.6 (8)	C17—C16—H16A	109.9 (9)
N1—C9—H9B	108.0 (7)	C15—C16—H16A	107.7 (9)
C10—C9—H9B	109.9 (7)	C17—C16—H16B	109.9 (9)
H9A—C9—H9B	108.9 (10)	C15—C16—H16B	109.6 (9)
C11—C10—C9	112.57 (9)	H16A—C16—H16B	105.9 (13)
C11—C10—H10A	111.3 (8)	C16—C17—H17A	109.7 (10)
C9—C10—H10A	109.3 (8)	C16—C17—H17B	111.0 (10)
C11—C10—H10B	109.4 (7)	H17A—C17—H17B	106.5 (15)
C9—C10—H10B	109.0 (7)	C16—C17—H17C	112.2 (9)
H10A—C10—H10B	104.9 (11)	H17A—C17—H17C	108.7 (14)
C12—C11—C10	113.04 (9)	H17B—C17—H17C	108.6 (14)
C12—C11—H11A	110.9 (8)		
			
C8—N1—C1—C2	178.81 (10)	C1—C6—C7—C8	−1.98 (10)
C9—N1—C1—C2	−6.62 (15)	C1—N1—C8—O2	177.89 (11)
C8—N1—C1—C6	−0.19 (11)	C9—N1—C8—O2	3.47 (18)
C9—N1—C1—C6	174.37 (9)	C1—N1—C8—C7	−1.07 (11)
C6—C1—C2—C3	−2.13 (15)	C9—N1—C8—C7	−175.49 (9)
N1—C1—C2—C3	178.96 (9)	O1—C7—C8—O2	2.55 (17)
C1—C2—C3—C4	0.59 (16)	C6—C7—C8—O2	−177.09 (11)
C2—C3—C4—C5	1.19 (18)	O1—C7—C8—N1	−178.47 (10)
C3—C4—C5—C6	−1.41 (17)	C6—C7—C8—N1	1.89 (11)
C4—C5—C6—C1	−0.11 (15)	C8—N1—C9—C10	103.80 (12)
C4—C5—C6—C7	179.33 (11)	C1—N1—C9—C10	−69.94 (12)
C2—C1—C6—C5	1.95 (15)	N1—C9—C10—C11	167.45 (9)
N1—C1—C6—C5	−178.98 (9)	C9—C10—C11—C12	−173.60 (9)
C2—C1—C6—C7	−177.62 (9)	C10—C11—C12—C13	175.57 (9)
N1—C1—C6—C7	1.45 (11)	C11—C12—C13—C14	−178.95 (10)
C5—C6—C7—O1	−1.08 (19)	C12—C13—C14—C15	177.47 (10)
C1—C6—C7—O1	178.42 (11)	C13—C14—C15—C16	−179.94 (10)
C5—C6—C7—C8	178.52 (11)	C14—C15—C16—C17	174.71 (10)

### Hydrogen-bond geometry (Å, º)

**Table:**

*D*—H···*A*	*D*—H	H···*A*	*D*···*A*	*D*—H···*A*
C2—H2···O2^i^	0.992 (13)	2.412 (13)	3.3737 (13)	163.3 (10)
C3—H3···O1^ii^	0.997 (14)	2.454 (15)	3.2734 (14)	139.0 (11)
C9—H9*B*···O1^i^	0.994 (13)	2.546 (13)	3.5012 (13)	161.0 (10)
C17—H17*B*···O2^iii^	0.98 (2)	2.49 (2)	3.3941 (17)	153.3 (15)

Symmetry codes: (i) *x*, −*y*+3/2, *z*−1/2; (ii) *x*, −*y*+1/2, *z*−1/2; (iii) −*x*+1, −*y*+2, −*z*+1.
